# The effect of temperature, humidity, precipitation and cloud coverage on the risk of COVID-19 infection in temperate regions of the USA—A case-crossover study

**DOI:** 10.1371/journal.pone.0273511

**Published:** 2022-09-15

**Authors:** Moritz K. Jansson, Shelby Yamamoto

**Affiliations:** 1 Institute of Medical Microbiology, Virology and Hygiene, University Medicine Rostock, Rostock, Germany; 2 School of Public Health, Edmonton Clinic Health Academy, University of Alberta, Edmonton, Alberta Canada; 3 Faculty of Epidemiology and Population Health, London School of Hygiene and Tropical Medicine, London, United Kingdom; Universite du Quebec a Montreal, CANADA

## Abstract

**Background:**

Observations based on the spread of SARS-CoV-2 early into the COVID-19 pandemic have suggested a reduced burden in tropical regions leading to the assumption of a dichotomy between cold and dry and wet and warm climates.

**Objectives:**

Analyzing more than a whole year of COVID-19 infection data, this study intents to refine the understanding of meteorological variables (temperature, humidity, precipitation and cloud coverage) on COVID-19 transmission in settings that experience distinct seasonal changes.

**Methods and findings:**

A time stratified case-crossover design was adopted with a conditional Poisson model in combination with a distributed lag nonlinear model to assess the short-term impact of mentioned meteorological factors on COVID-19 infections in five US study sites (New York City (NYC); Marion County, Indiana (MCI); Baltimore and Baltimore County, Maryland (BCM); Franklin County, Ohio (FCO); King County, Washington (KCW)). Higher-than-average temperatures were consistently associated with a decreased relative risk (RR) of COVID-19 infection in four study sites. At 20 degrees Celsius COVID-19 infection was associated with a relative risk of 0.35 (95%CI: 0.20–0.60) in NYC, 1.03 (95%CI:0.57–1.84) in MCI, 0.34 (95%CI: 0.20–0.57) in BCM, 0.52 (95%CI: 0.31–0.87) in FCO and 0.21 (95%CI: 0.10–0.44) in KCW. Higher-than-average humidity levels were associated with an increased relative risk of COVID-19 infection in four study sites. Relative to their respective means, at a humidity level of 15 g/kg (specific humidity) the RR was 5.83 (95%CI: 2.05–16.58) in BCM, at a humidity level of 10 g/kg the RR was 3.44 (95%CI: 1.95–6.01) in KCW.

**Conclusions:**

The results of this study suggest opposed effects for higher-than-average temperature and humidity concerning the risk of COVID-19 infection. While a distinct seasonal pattern of COVID-19 has not yet emerged, warm and humid weather should not be generally regarded as a time of reduced risk of COVID-19 infections.

## Background

Climatic factors have been linked to the spread of viral respiratory pathogens such as the influenza virus, which shows a recurring seasonal pattern [1]. Consequently, it has been hypothesized that the spread of the SARS-Cov-2 virus, responsible for the ongoing COVID-19 pandemic, might be influenced by meteorological variables. A strong interest concerning the influence of meteorological variables on COVID-19 infections—notably temperature and humidity- led to the rapid accumulation of a body of research in the early phase of the COVID-19 pandemic [2–17]. In their quest to reach timely conclusions to inform public health policy researchers had to make use of what little data was available.

### Observations on the effect of temperature and humidity on COVID-19 so far

Observations based on the spread of SARS-CoV-2 early into the COVID-19 pandemic seemed to suggest a reduced COVID-19 burden in tropical regions leading to the assumption of a dichotomy between cold and dry and wet and warm climates and a resulting synergy between humidity and temperature on COVID-19 infection [5]. These assumptions were partly supported by early exploratory studies [18], however there was some degree of heterogeneity. One study analyzing cases in New South Wales, Australia between January and May 2020 did not identify a relationship between COVID-19 and temperature but found a 7–8% increase between COVID-19 cases and every 1% decrease in relative humidity [4].

### Possible underlying causes of the effect of meteorological parameters on COVID-19

Arguably, the focus on temperature and humidity results from observations of direct effects on virus stability and analogous epidemiological findings consistent with other respiratory pathogens. For instance, transmissible gastroenteritis virus (TGEV) and mouse hepatitis virus (MHV) used as surrogates to assess survival of SARS-CoV on stainless steel surfaces were degraded more rapidly at 20°C than at 4°C at all humidity levels. Survival was greater at low and high humidity levels (20% and 80%) than at moderate levels (50%) [19]. Influenza epidemics show a distinct seasonal pattern in temperate regions and influenza survival and transmission has been shown to be modulated by absolute humidity [20]. However, not only determinants of virus survival might play a role in transmission. Meteorological variables such as temperature, windspeed and rainfall have been demonstrated to influence the behavior of the human host of SARS-CoV-2 which, indirectly, might result in changes in COVID-19 related outcomes [21]. These behavioral patterns might result in consistent changes in COVID-19 transmission dynamics. One study correlated the effect of various meteorological variables on COVID-19 related outcomes in all provinces of China and found precipitation to be positively associated with COVID-19 related deaths in Fujian and negatively associated with COVID-19 cases in Shanghai [22]. One study that was restricted to the city of Oslo found precipitation to be negatively associated with COVID-19 [2] while others found no association [4, 6, 7]. One study correlated cloud coverage amongst other climatic variables with COVID-19 cases and COVID-19 related mortality but found no association [8].

### The objectives of the present study

Many studies so far have been limited by only capturing a short duration of the COVID-19 pandemic and therefore represent a limited range and frequency of meteorological parameters in environments experiencing seasonal changes. In the light of the ongoing COVID-19 pandemic, the present study intents to further refine knowledge on the effect of meteorological variables on COVID-19 transmission in the temperate climate zone. The study focuses on highly populated urban and county level areas in temperate environments of the USA to investigate the effect of temperature, humidity, precipitation, and cloud coverage on the number of reported COVID-19 cases.

## Materials and methods

### Data collection

#### COVID-19 case data

**Fig 1:**
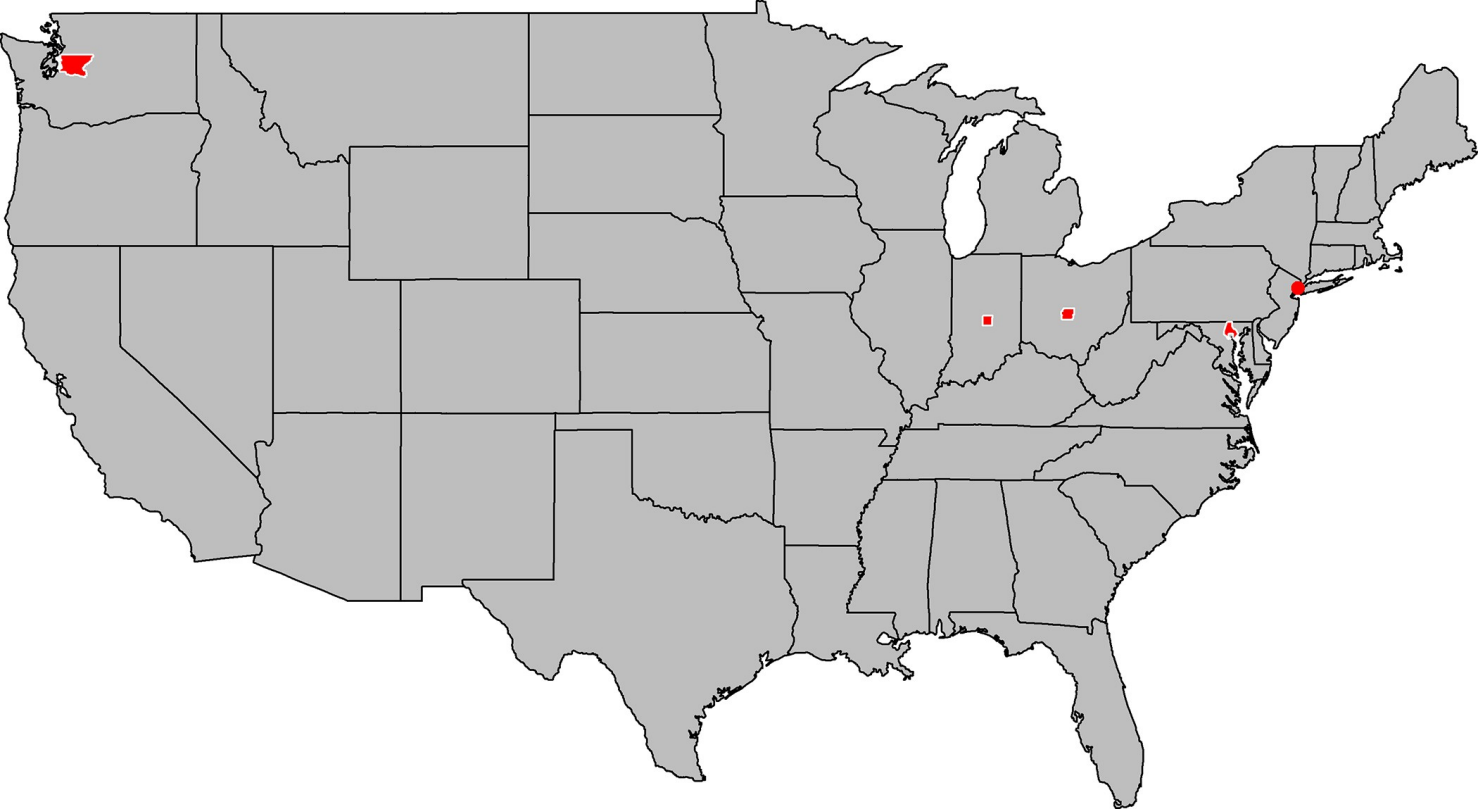
Study sites selected for analysis. Study sites are marked red. From left to right: KCW: King County, Washington (including Seattle); MCI: Marion County, Indiana (including Indianapolis); Franklin County, Ohio (including Columbus); BCM: Baltimore County, Maryland (including Baltimore); NYC: New York City.

Data on reported COVID-19 infections were collected from fully anonymized, publicly available US databases that have been described as recording the date of COVID-19 diagnosis, not the date reported to health authorities [23] (Table 1). A case was defined as a COVID-19 infection reported. The study period was defined from the point of time at which more than zero COVID-19 cases were first reported on each consecutive day at the respective study sites until the 01^st^ of May 2021. Study sites were selected to be within the temperate regions according to the Köppen–Geiger climate classification system characterized by wider temperature ranges and distinct seasonal changes [24] (Fig 1).

**Table 1:** Origin of data on daily COVID-19 infections for different study sites.

Study Site	Data Source	Study period	URL
New York City (NYC)	NYC Health Department	03/03/2020-5/1/2021	https://github.com/nychealth/coronavirus-data
Marion County, Indiana (MCI)	Indiana Government	03/17/2020-05/01/2021	https://www.coronavirus.in.gov/
Baltimore and Baltimore County, Maryland (BCM)	Maryland Government	03/15/2020-05/01/2021	https://coronavirus.maryland.gov/search?collection=Dataset
Franklin County, Ohio (FCO)	Ohio Department of Health	02/28/2020-05/01/2021	https://coronavirus.ohio.gov/wps/portal/gov/covid-19/dashboards/overview
King County, Washington (KCW)	King County Government	03/15/2021-05/01/2021	https://kingcounty.gov/depts/health/covid-19/data.aspx

#### Climate data

ERA5 is the fifth-generation reanalysis for the global climate and weather by the European Centre for Medium-Range Weather Forecasts (ECMWF). ERA5 provides hourly estimates for atmospheric, ocean-wave and land-surface quantities which are made publicly available via the Climate Data Store (CDS) (https://cds.climate.copernicus.eu/#!/home) [25]. It has been shown that ERA5 reanalysis data are reliable for use in environmental epidemiological analyses [26]. Data on meteorological variables can be retrieved from CDS defined as tiles at 0.25 degrees (27.75 kilometers) horizontal resolution. Central coordinates of each location were identified and the tile closest to these coordinates was chosen to represent climate variables of the respective location. Data on hourly temperature (°C), specific humidity (g/kg), total precipitation (mm) and cloud coverage (0–100%), were retrieved from CDS. Specific humidity was chosen as it is less influenced by temperature than absolute or relative humidity, thereby reducing correlation between the two variables.

#### COVID-19 government response data

A stringency index by the Oxford COVID-19 Government Response Tracker (https://www.bsg.ox.ac.uk/research/research-projects/covid-19-government-response-tracker) composed of nine individual component indicators (school closing, workplace closing, cancel public events, restrictions on gatherings, close public transport, stay at home requirements, restrictions on internal movement, international travel controls and public information campaigns) was used to account for possible confounding by behavioral changes due to federally imposed restrictions. The index represents the strictness of ‘lockdown style’ policies at US state level and ranges from zero to 100. To facilitate integration into the model, the daily values of the index during the study period were broken into four equally sized stringency index categories (SIC) (1: 0–25, 2: 26–50, 3: 51–75, 4: 76–100).

### The model

#### Case-crossover model

A time stratified case-crossover design was chosen with a conditional Poisson model [27]. The case-crossover design was used as it provides an efficient way to control for confounding factors that are constant within one person by using the history of COVID-19 cases within the study period as control periods. In the case-crossover design all cases observed during the study period are included, while non-cases do not contribute to the analysis. Effects of exposures are accounted for in the analysis by defining a time window that starts after the exposure of interest and which is chosen in accordance with the estimated duration of the effect. The exposed person-time until the event can be estimated by multiplying the frequency of exposure by the time of the effect window while unexposed person-time is calculated by subtraction of the exposed person time from the total person time [28]. As COVID-19 infections have been shown to follow a seven-day cycle, possibly due to delayed reporting or testing habits, time was stratified by calendar month to account for seasonal effects and day of the week to account for weekly cycles [23].

#### Distributed lag nonlinear model (DLNM)

Integration of the time dimension of the exposure-response relationship was facilitated by a distributed lag nonlinear model (DLNM) using the dlnm R package. DLNMs are capable of modelling non-linear associations between exposures and outcomes and delayed effects. DLNMs can also be applied to linear relationships or categorized predictor variables [29]. Natural cubic splines were used to model the lagged effect of meteorological variables on COVID-19 infections. Spline knots were placed at equal spaces in the range of climate variables to allow sufficient flexibility at both ends of their distributions and at equal intervals on the log scale of lags to allow for more flexible lag effects over shorter periods as previously described [30]. The lag period was set between 2–14 days after the exposure in accordance with estimates of the incubation period of SARS-CoV-2 [31]. Intercepts for precipitation were set at zero, for temperature, specific humidity, and cloud coverage, mean values were used.

#### Model adjustments to account for time series regression of an infectious disease

As rapid changes of daily case counts and clustering within strata was expected, overdispersion was accounted for by using a quasi-Poisson distribution model. For residuals from time series regression models of infectious diseases, autocorrelation is often strong as the number of cases occurring at time t will be directly dependent on the size of the recent infectious population[32]. After analyses of autocorrelation using the NYC dataset, lagged disease counts were included as a variable to reduce autocorrelation.

### Data analysis

The effect of temperature and humidity was first analyzed individually, then adjusted for humidity and temperature, respectively (formula 1).

As precipitation depends on cloud coverage, precipitation was considered to lie on the causal pathway between cloud coverage and COVID-19 infections and it was not considered a confounder. Based on this consideration, the effect of the two variables was assessed separately. After conduction of sensitivity analysis, only temperature but not humidity was included as a confounding factor (formula 2).

The possibility of a threshold below which precipitation mediates no human behavioral change and above which humans change their behavior abruptly was considered. Days with precipitation levels between 0–10 mm has been classified as light in previous studies and was considered unlikely to result in meaningful behavioral changes [33]. Above 10 mm of precipitation a behavioral change (e.g., staying indoors rather than leaving outside) was considered. It was hypothesized that an increase of indoor clustering due to precipitation would likely result in an increase of COVID-19 infections as most transmission events are associated with indoor environments [34]. For this part of the analysis, the model was adapted by dividing precipitation into two strata according to the threshold level.

Applying the above steps and considering results from the sensitivity analysis, the following formulas were used:

$$Y_{t}∼Poisson(\mu_{t})$$

$$Log\;(\mu_{t})=\alpha +\beta_{1}T_{t,l}+\beta_{2}H_{t,l}+\lambda {Strata}_{t}+\eta {SIC}_{t}+\upsilon Y_{t-n}$$ (1)

$$Log\;(\mu_{t})=\alpha +\beta_{1}P_{t,l}|{CC}_{t,l}+\beta_{2}T_{t,l}+\lambda {Strata}_{t}+\eta {SIC}_{t}+\upsilon Y_{t-n}$$ (2)

where t is the day of the observation; Y_t_ is the observed daily COVID-19 case counts on day t; α is the intercept; T_t,l_ /H _t,l_ /P_t,l_ /CC_t,l_ are matrices obtained by applying the DLNM to temperature/humidity/precipitation/cloud coverage, β_1_ and β_2_ are the vectors of coefficients for T_t,l_ /H _t,l_ /P_t,l_ /CC_t,l_ and l are the lag days. Strata_t_ is a categorical variable of the year, calendar month and day of the week used to control for season and trends, and λ is the vector of coefficients. SIC_t_ is a categorical variable of the stringency index on day t, and η is the vector of coefficients. Y_t-n_ is the COVID-19 case count of day t–n, and υ is the coefficient.

The DLNM was used to predict the effects (β) and standard errors for combinations of temperature/humidity precipitation/cloud coverage levels and lags [30]. Under the rare-disease assumption, we equated the odds ratios with the relative risk of COVID-19 infection. Relative risks with 95% confidence intervals (95% CIs) are reported. We adapted the model used by Runkle et al who used a case-crossover design and DLNM to study the effects of meteorological variables on COVID-19 based on a model by Guo et al. [9, 30].

#### Sensitivity analysis

We assessed model fit using quasi-Akaike Information criterion (QAIC). Model fit under varying degrees of freedom for meteorological variables (2–9) and lag periods (3–5) using the NYC dataset was compared. The lowest absolute QAIC value retrieved for a given combination of parameters was considered as the combination resulting in the best model fit.

Residual autocorrelation was assessed using the NYC dataset by determining model residuals and using the partial autocorrelation function (PACF). Lagged autocorrelation of residuals for the past 20 days was considered. The day (t-n) that showed the highest residual autocorrelation was identified. The effect of including the COVID-19 case count/logged COVID-19 case count/model residual of t-n as additional model variables on residual autocorrelation and model fit was assessed. The variable leading to the greatest reduction in autocorrelation was chosen for inclusion in the final model.

The effect of possible confounding variables (temperature, humidity as well as categorized stringency index [CSI] and autocorrelation variables [AC]) was investigated by comparing model fit before and after inclusion as determined by corresponding QAIC values. The models resulting in the lowest QAIC values were determined and selected as final models.

Data were analyzed using statistical software package Rstudio version 1.2.5033. Figures were created using R packages ggplot2, dlnm and plotly.

### Results

#### Explorative analysis

Five study sites were selected for analysis: New York City (NYC), Marion County, Indiana including its county seat Indianapolis (MCI), Baltimore County, Maryland including the city of Baltimore (BCM), Franklin County, Ohio including its county seat Columbus (FCO) and King County, Washington including its county seat Seattle (KCW). Population size varied at each site with NYC being the most populous site and MCI the least. The highest percentage of reported cases compared to total population was observed in MCI (10.2%). The lowest percentage was observed in KCW (4.6%). Mean temperature was relatively similar between study sites. The lower range of temperature in MCI and FCO was considerably lower than for the other study sites (Table 2).

**Table 2:** Descriptive analysis of meteorological variables at different study sites during the study period.

Site	population	ERA5 coordinates (Long.,Lat.)	Study period	Total cases	Temperature (°C)	Humidity (g/kg)	Precipitation (mm/day)	cloud coverage (0–1)
NYC	8,804,190	-74.0, 40.75	03/03/2020-5/1/2021	770,598	12.5 (-6.2–29.9)	6.7 (0.9–17.2)	3.3 (0–45.4)	0.57 (0–1)
MCI	964,582	-86.25, 39.75	03/17/2020-05/01/2021	98,570	12.2 (-12.2–28.8)	6.9 (0.9–17.6)	2.6 (0–47.2)	0.59 (0–1)
BCM	1,463,564	-76.5, 39.25	03/15/2020-05/01/2021	114,716	13.9 (-0.1–31.5)	7.5 (1.0–18.9)	3.6 (0–69.6)	0.58 (0–1)
FCO	1,316,756	-83.0, 40.0	02/28/2020-05/01/2021	124,559	11.6 (-12.1–28.8)	6.8 (1.1–17.1)	3.1 (0–41.5)	0.61 (0–1)
KCW	2,269,675	-122.25,47.5	03/15/2021-05/01/2021	104,003	11.2 (-2.1–24.9)	6.1 (1.5–11.9)	3.8 (0–38.3)	0.65 (0–1)

NYC: New York City; MCI: Marion County, Indiana (including Indianapolis); BCM: Baltimore County, Maryland (including Baltimore), Franklin County, Ohio (including Columbus), KCW: King County, Washington (including Seattle). For temperature, humidity, precipitation and cloud coverage mean values and ranges over the study period are shown.

**Fig 2:**
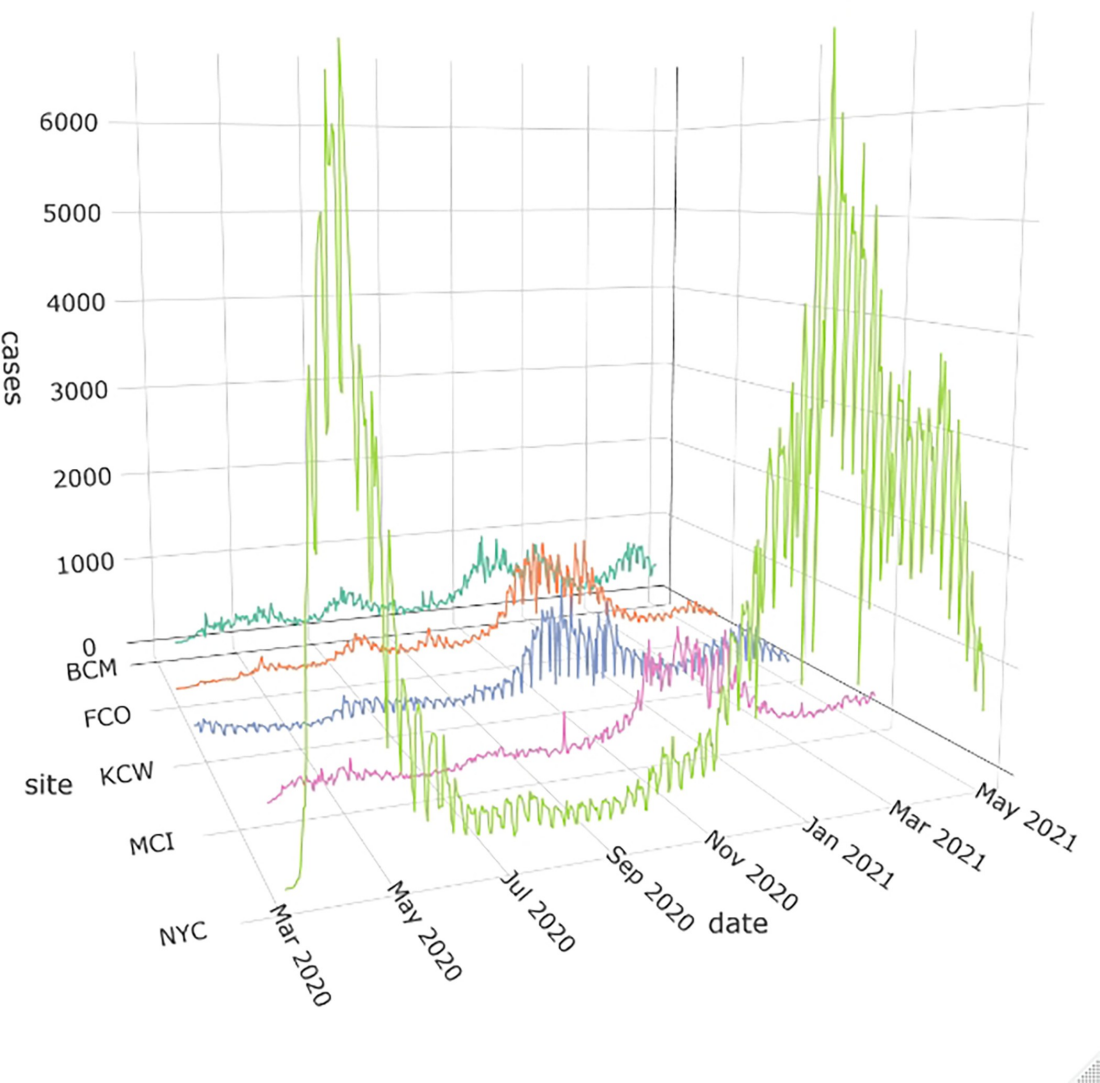
COVID-19 cases reported at the study sites over the course of the study period. NYC: New York City; MCI: Marion County, Indiana (including Indianapolis); BCM: Baltimore County, Maryland (including Baltimore), Franklin County, Ohio (including Columbus), KCW: King County, Washington (including Seattle).

Reported COVID-19 cases at different study sites showed a wave pattern (Fig 2). Temperature and specific humidity were strongly correlated at all study sites (Spearman correlation coefficient for NYC: 0.92). There was also pronounced correlation observable between cloud coverage and precipitation (Spearman correlation coefficient for NYC: 0.65) (S1 Fig).

#### Sensitivity analysis

Setting the degrees of freedom for the lag period to three tended to minimize QAIC values. Varying the degrees of freedom for meteorological variables beyond four did not result in strong reductions of QAIC values. Prioritizing model parsimony, four degrees of freedom were adopted for all meteorological variables (S1 Table).

Residual autocorrelation was strongest at lag of day 1 for the precipitation and cloud coverage model (S2A and S3A Figs). Including the COVID-19 case count of the previous day (Y_t-1_) (S2B and S3B Figs) or the logged COVID-19 case count of the previous day (log(_Yt-1_)) (S2C and S3C Figs) resulted in comparable reductions of autocorrelation among model residuals. The inclusion of lagged residuals also led to some reduction of autocorrelation, but the effect was considerably less pronounced. Using Y_t-1_ led to a greater decrease of the QAIC values than log(Y_t-1_) for precipitation and cloud coverage and was therefore included in the model.

Model fit was analyzed for all study sites under inclusion of different confounding factors added to the temperature and humidity model. With the exception of site FCO, temperature seemed to be the more robust predictor of COVID-19 infections, according to model fit (Table 3). QAIC values tended to show a decease with the inclusion of additional confounding factors. For the investigation of the effect of temperature and humidity on COVID-19 infections, all confounding factors presented in Table 3 were included.

**Table 3:** QAIC values according to nested models with temperature and humidity plus confounders.

location	T^a^	H^b^	T+H	T+H+CSI^c^	T+H+AC^d^	T+H+CSI+AC
NYC	43035.71	48325.72	43392.67	36572.4	30311.26	28238.20
MCI	10384.65	10732.08	10312.93	10118.26	8475.963	8397.971
BCM	9306.779	10922.44	8897.512	8964.923	8233.52	8302.103
FCO	10312.78	10202.40	9898.85	9975.87	8199.96	8259.05
KCW	10927.0	10973.56	10053.12	9933.36	9284.54	9235.27

^a^ T: Temperature

^b^ H: Humidity

^c^CSI: categorized stringency index

^d^AC: autocorrelation variable (Y_t-1_). Model fit for temperature and humidity was assessed alone and with additional parameters. The lowest absolute QAIC value retrieved for a given combination of parameters was considered as the combination resulting in the best model fit.

For the precipitation and cloud coverage model inclusion of temperature and Y_t-1_ to account for autocorrelation, led to a large decrease in QAIC values. Inclusion of the categorized stringency index resulted in a relatively small decrease in QAIC values for most datasets. The inclusion of humidity led to an increase in some instances and a decrease in others with little changes to the overall results. Therefore, under considerations of model parsimony, humidity was not included in the final precipitation and cloud coverage models (Table 4).

**Table 4:** QAIC values returned depending on integration of variables into the precipitation and cloud coverage models respectively.

**location**	**precipitation**	**+T** ^**a**^	**T+CSI** ^**c**^	**+T+AC** ^**d**^	**+T+CSI+AC**	**+T+CSI+AC+H** ^**b**^
NYC	42225.89	38403.67	32840.70	28581.77	26436.33	27677.62
MCI	9584.99	9230.82	9202.78	8288.73	8184.46	8278.94
BCM	11027.15	8995.05	8910.18	8541.47	8472.394	8019.42
FCO	9055.84	8849.02	8908.83	8104.63	8163.96	8032.86
KCW	11118.53	10660.35	10641.34	9555.94	9577.73	8975.09
	**Cloud coverage**	**+T** ^**a**^	**T+CSI** ^**c**^	**+T+AC** ^**d**^	**+T+CSI+AC**	**+T+CSI+AC+H** ^**b**^
NYC	43893.64	38141.92	34619.05	29174.88	27397.62	28385.80
MCI	10304.12	9706.42	9554.07	8359.30	8266.08	8354.63
BCM	10849.23	8832.65	8796.05	8341.90	8324.50	8315.78
FCO	9823.70	9873.81	9938.05	8082.31	8131.46	7917.56
KCW	10124.19	9599.97	9539.72	9119.41	9118.48	9000.64

^a^ T: Temperature

^b^ H: Humidity

^c^CSI: categorized stringency index

^d^AC: autocorrelation variable (Y_t-1_). Model fit for precipitation and cloud coverage was assessed alone and with additional parameters. The lowest absolute QAIC value retrieved for a given combination of parameters was considered as the combination resulting in the best model fit.

#### Relationship between temperature and humidity and COVID-19 infections

**Fig 3:**
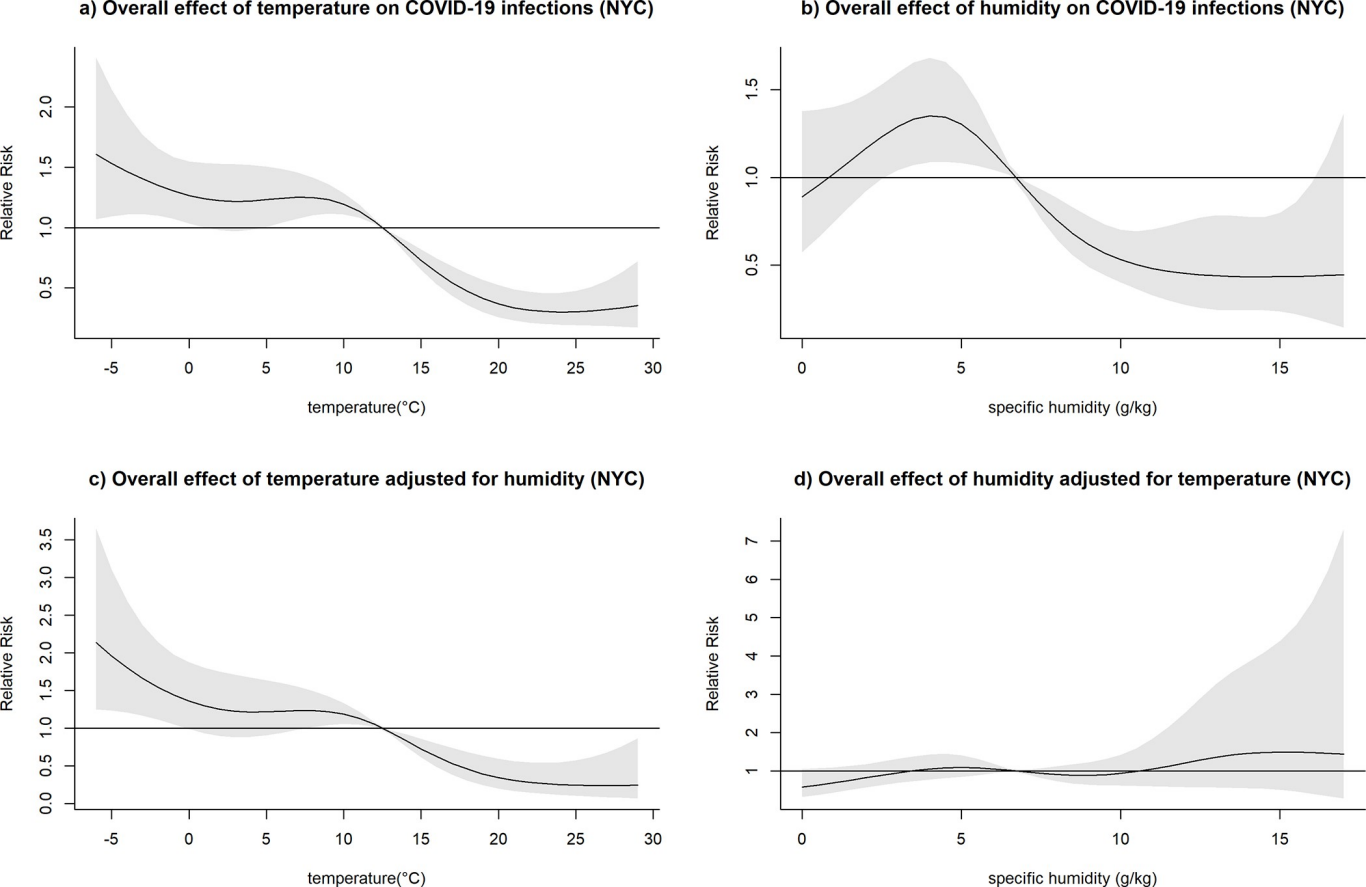
Overall effect of temperature and humidity on COVID-19 infections in NYC. The relative risk of COVID-19 infection was plotted across the range of temperature/humidity to show the a) Unadjusted effect of temperature; b) Unadjusted effect of humidity; c) Effect of temperature adjusted for humidity; d) Effect of humidity adjusted for temperature.

**Fig 4:**
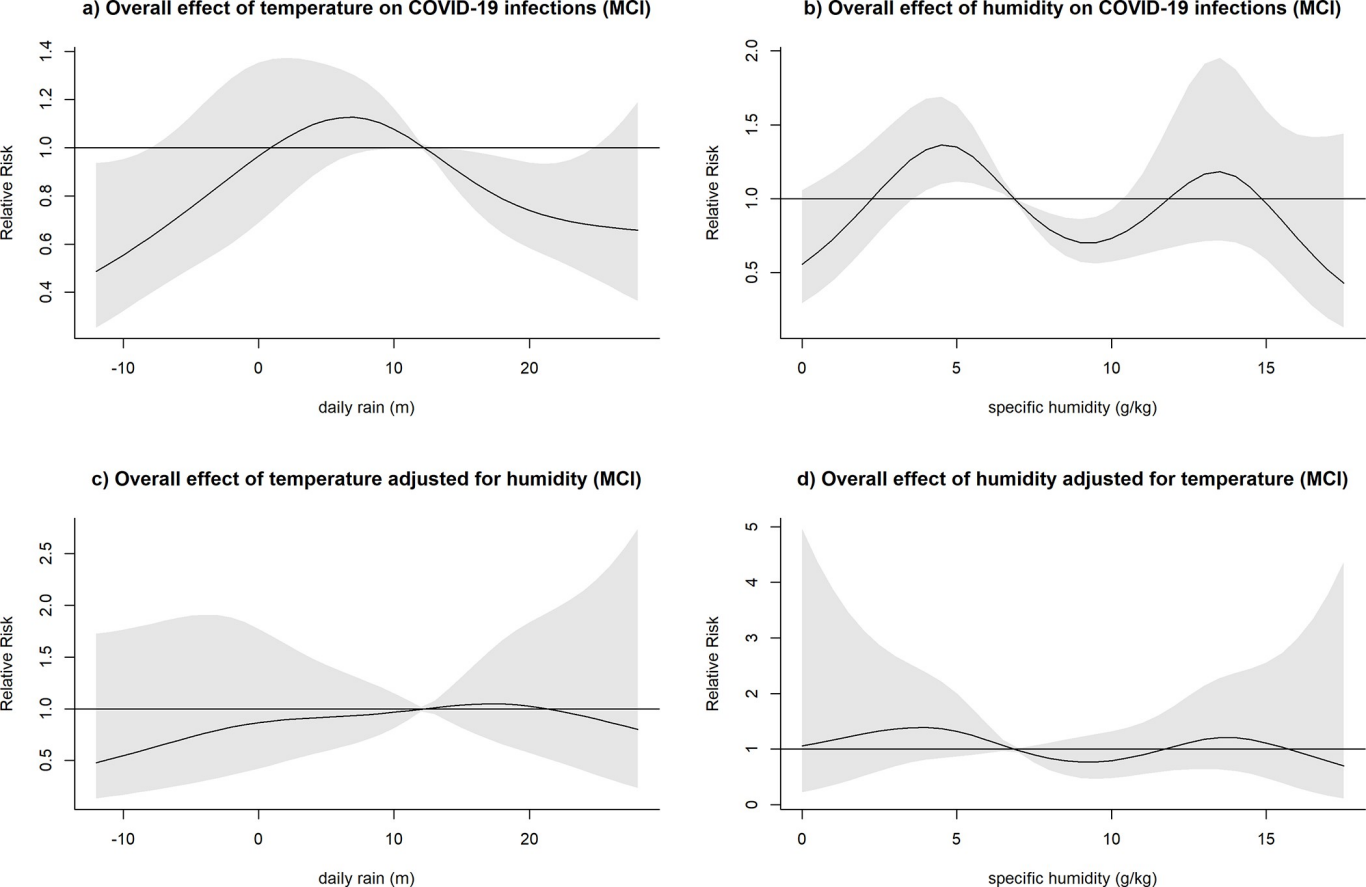
Overall effect of temperature and humidity on COVID-19 infections in Marion County, Indiana (MCI). The relative risk of COVID-19 infection was plotted across the range of temperature/humidity to show the a) Unadjusted effect of temperature; b) Unadjusted effect of humidity; c) Effect of temperature adjusted for humidity; d) Effect of humidity adjusted for temperature.

**Fig 5:**
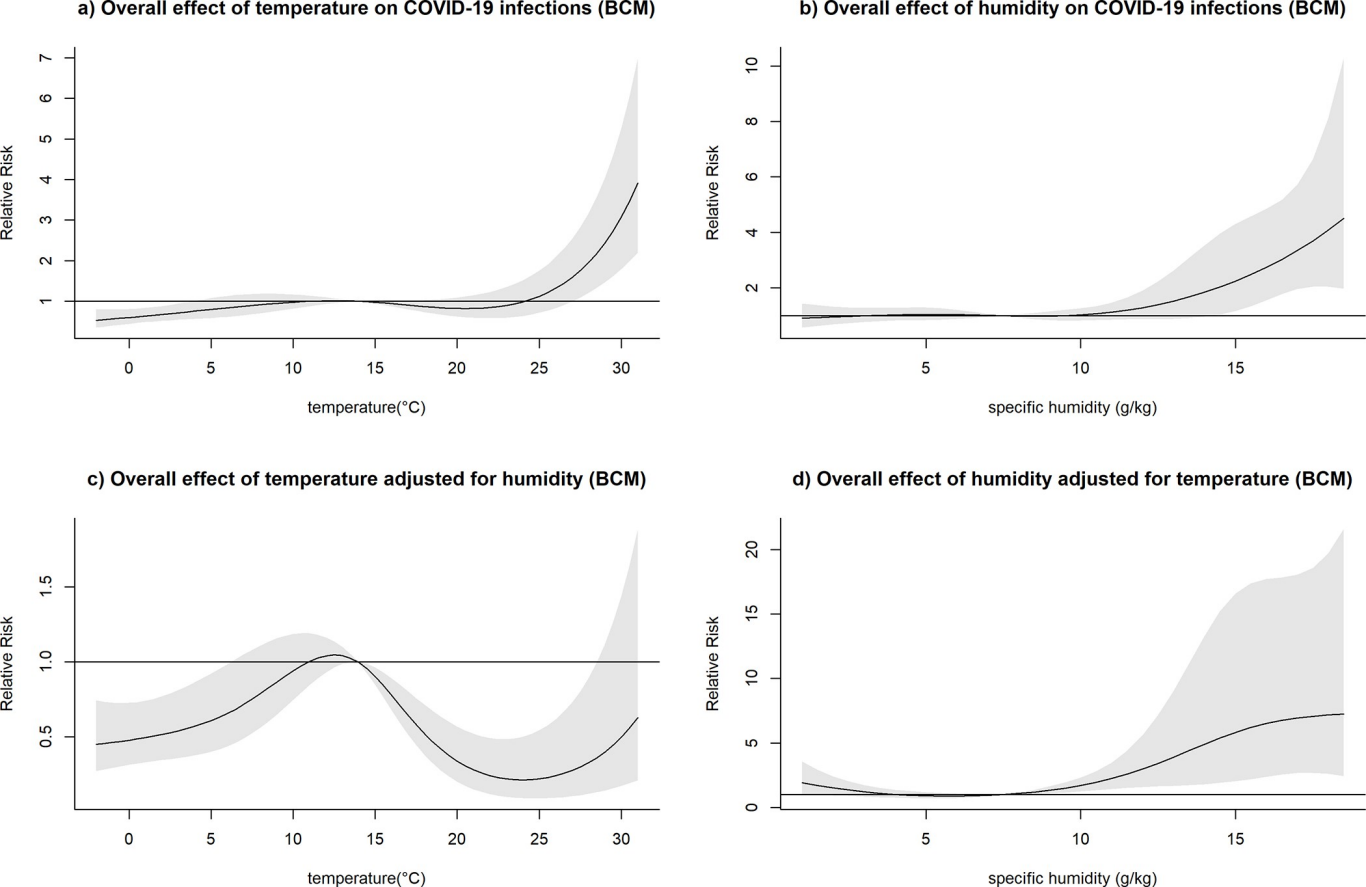
Overall effect of temperature and humidity on COVID-19 infections in Baltimore and Baltimore County, Maryland (BCM). The relative risk of COVID-19 infection was plotted across the range of temperature/humidity to show the a) Unadjusted effect of temperature; b) Unadjusted effect of humidity; c) Effect of temperature adjusted for humidity; d) Effect of humidity adjusted for temperature.

**Fig 6:**
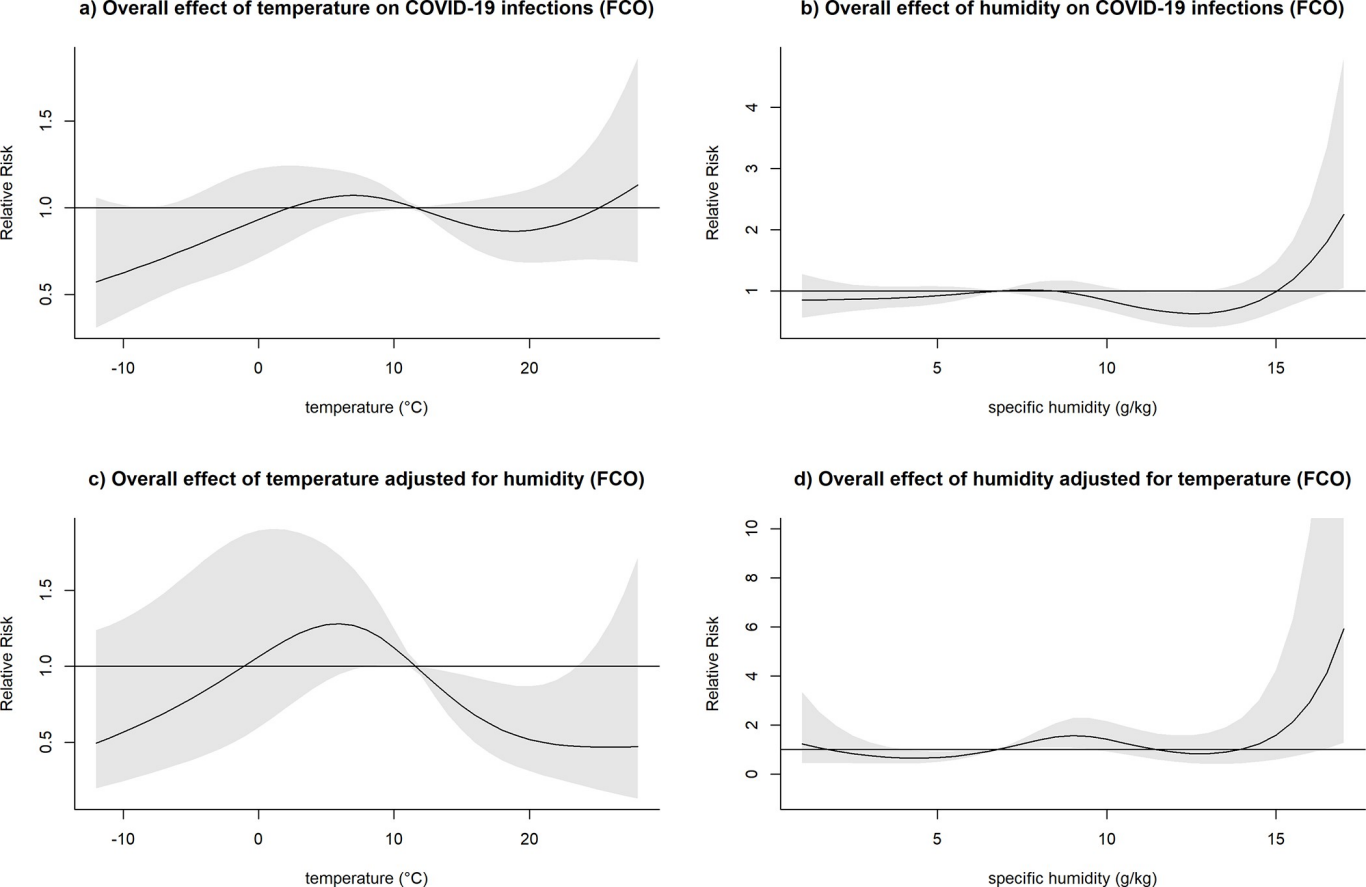
Overall effect of temperature and humidity on COVID-19 infections in Franklin County, Ohio (FCO). The relative risk of COVID-19 infection was plotted across the range of temperature/humidity to show the a) Unadjusted effect of temperature; b) Unadjusted effect of humidity; c) Effect of temperature adjusted for humidity; d) Effect of humidity adjusted for temperature.

**Fig 7:**
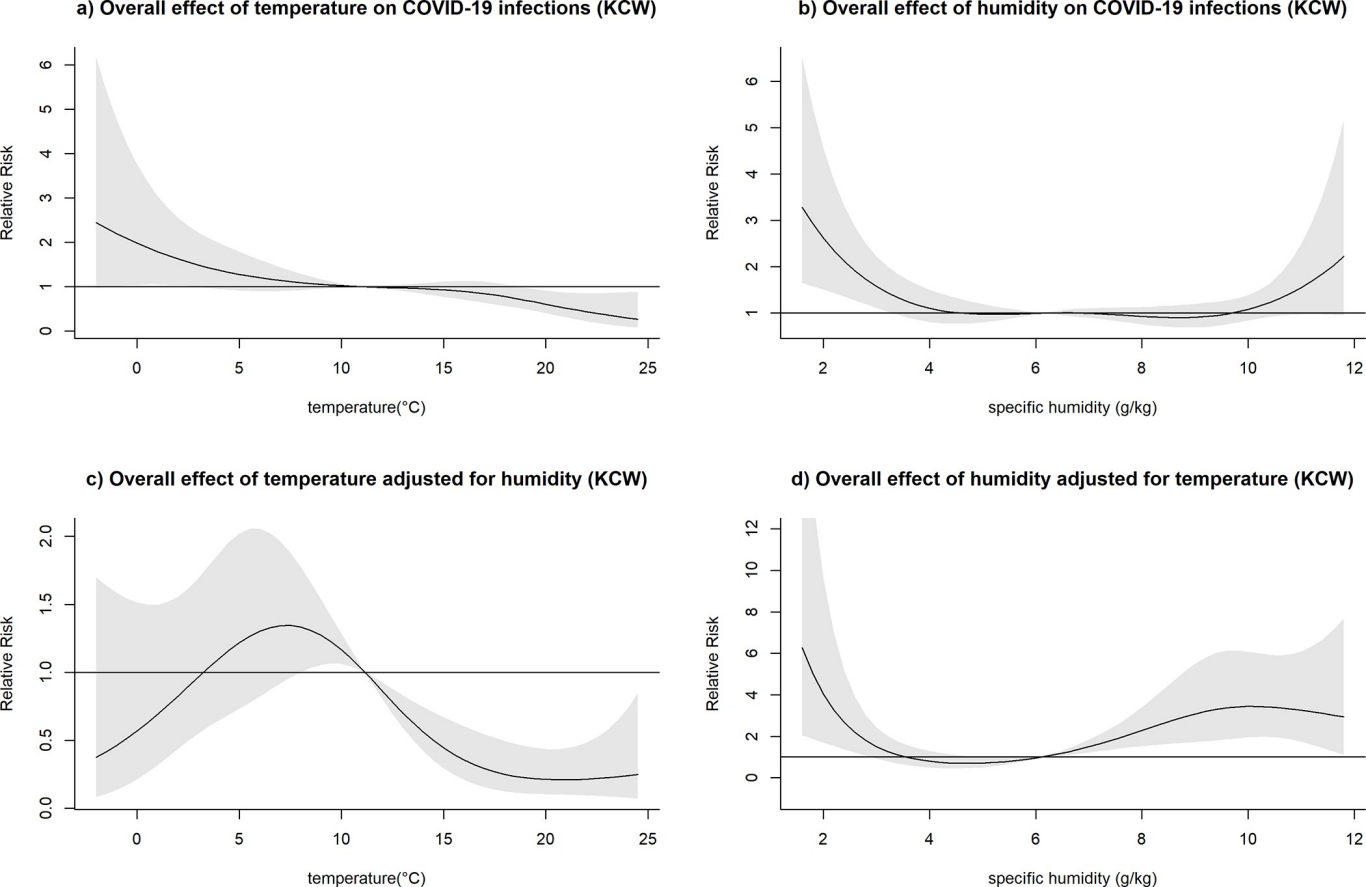
Overall effect of temperature and humidity on COVID-19 infections in Seattle and King County, Washington (KCW). The relative risk of COVID-19 infection was plotted across the range of temperature/humidity to show the a) Unadjusted effect of temperature; b) Unadjusted effect of humidity; c) Effect of temperature adjusted for humidity; d) Effect of humidity adjusted for temperature.

After adjusting for humidity, temperatures higher than the respective mean were consistently associated with a decreased relative risk of COVID-19 infection across all locations, with the exception of MCI (Figs 3–7). At the 20 degrees Celsius threshold, COVID-19 infections were associated with a relative risk of 0.35 (95%CI: 0.20–0.60) in NYC, 1.03 (95%CI:0.57–1.84) in MCI, 0.34 (95%CI: 0.20–0.57) in BCM, 0.52 (95%CI: 0.31–0.87) in FCO, and 0.21 (95%CI: 0.10–0.44) in KCW. In MCI, a similar effect of temperature was observed (0.74 (95%CI: 0.58–0.94)) in unadjusted analyses, though this relationship attenuated significantly after adjusting for humidity (Fig 4A and 4C).

The effect of lower-than-average temperatures seemed less pronounced than higher than average temperatures and showed some variation between the study sites. In the case of BCM, FCO, and KCW, a moderate increase in the relative risk seemed to be followed by a decreased risk of COVID-19 transmission at low temperatures (Figs 5–7). The range of temperatures associated with an increased risk of COVID-19 was between 11 to 14°C for BAL, 3.5 to 11°C for KCW, -1 to 11°C for FCO. For NYC, a consistent increase in the relative risk was observable for the range of temperatures below the mean (-6.2 to 12.5) (Fig 3).

After adjusting for temperature, higher than average humidity levels were associated with an increased relative risk of COVID-19 infection across most study sites. The strongest effects were observable in BCM and KCW. In BCM, a humidity level of 15 g/kg was associated with an increased relative risk of COVID-19 infection of 5.83 (95%CI: 2.05–16.58) and in KCW, a humidity level of 10 g/kg was associated with a relative risk of 3.44 (95%CI: 1.95–6.01) relative to their respective mean values (Figs 5 and 7). Low levels of humidity seemed not to be associated with of COVID-19 infection, with the exception of KCW where an increased relative risk was observed.

Both humidity and temperature were linked. In BCM, where the effect of humidity was very strong, the effect of temperature on COVID-19 infections was inversed after adjusting for humidity (Fig 5). Similarly, in NYC, where the effect of temperature was very pronounced, the effect of humidity on COVID-19 infections was inversed after adjusting for temperature (Fig 3). Overall, in adjusted analysis effects of temperature and humidity became more uniform across the different study sites, suggesting that adjusted analysis is necessary to detect the underlying effects of those meteorological variables on COVID-19 infections.

After adjusting for temperature, confidence intervals for humidity tended to become wider, possibly as a result of a loss of precision due to multicollinearity between the two variables. However, the association between temperature and COVID-19 did not show a meaningful loss of precision after adjusting for humidity.

#### Relationship between precipitation and COVID-19 infection

**Fig 8:**
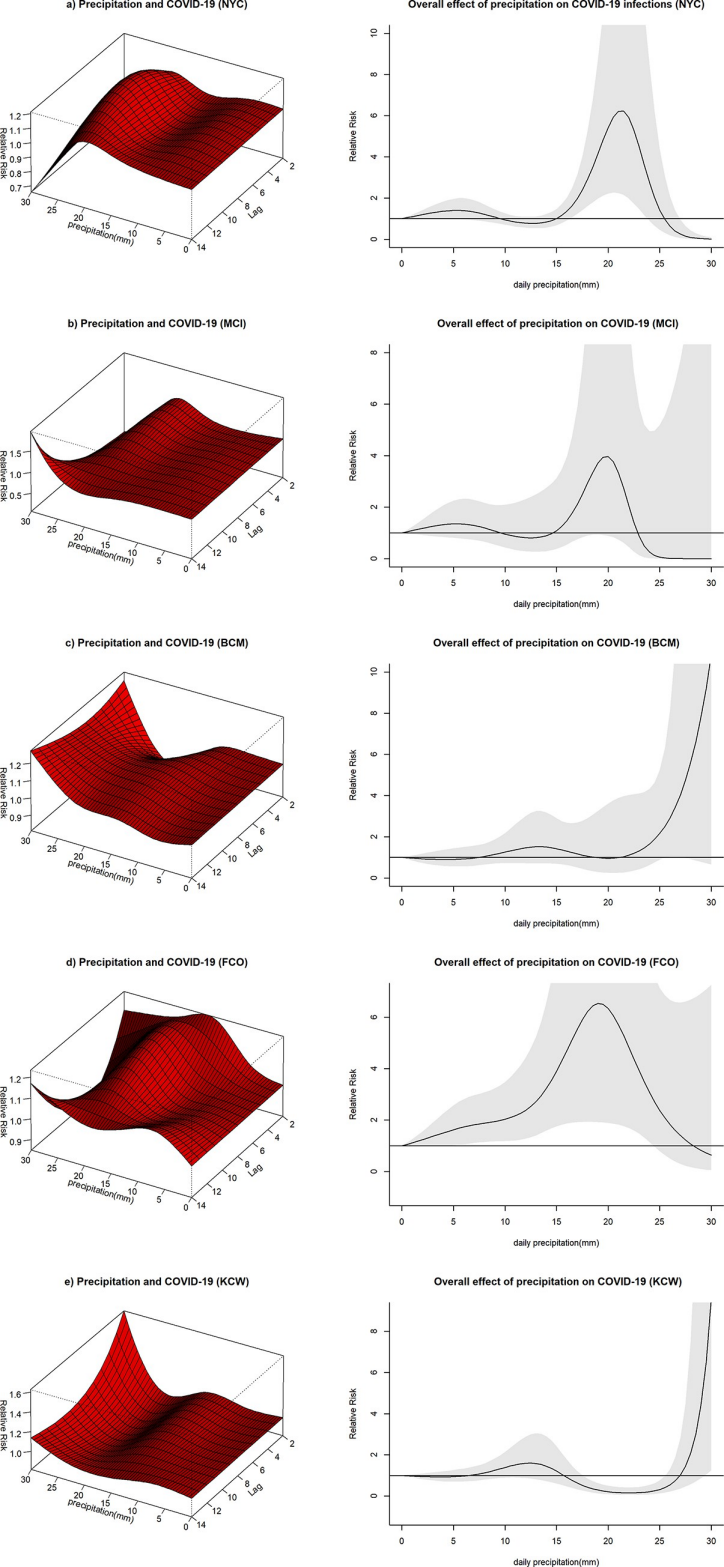
3D Plots of the effect of precipitation on COVID-19 infection risk across lags and overall plots of the effect of precipitation on COVID-19 infection risk excluding precipitation levels >30 mm daily for each study site. The surface of 3D plots visualizes the relative risk of COVID-19 infection (y-axis) for different levels of precipitation (x-axis, millimeters) across different lag times (z-axis, days). The overall effect plots provide an estimate of the combined relative risk of COVID-19 across the 14-day lag period with precipitation levels represented on the x-axis and the relative risk on the y-axis.

Heavy daily precipitation of >30 mm was rarely observed for the study locations during the study period (NYC: 5 days, MCI:4 days, BAL: 6 days, FRO:5 days, KCW: 2 days) and estimates were based on only a few outliers. Extreme precipitation levels seemed to be associated with an increased risk of COVID-19 (S4 Fig). Analyses were repeated after excluding precipitation levels of more than 30 mm (Fig 8).

With these outliers removed, the trend of an initial increased risk of COVID-19 infection remained, with a variably pronounced increase of risk starting between 10 to 15 mm of precipitation at the different study sites. However, the association was statistically significant only for NYC and FCO and was followed by a decrease in risk of COVID-19 infection across all study locations with the exception of BCM. In the case of KCW, the initial increase of risk was considerably smaller than the subsequent reduction in risk. Taking the overall effect of COVID-19 infection as well as the gradual changes of the effect across lags (3D plot) into consideration, there seemed to be some similarities in trends among NYC, MCI and FCO (Fig 8A, 8B and 8D) and BCM and KCW (Fig 8C and 8E).

**Fig 9:**
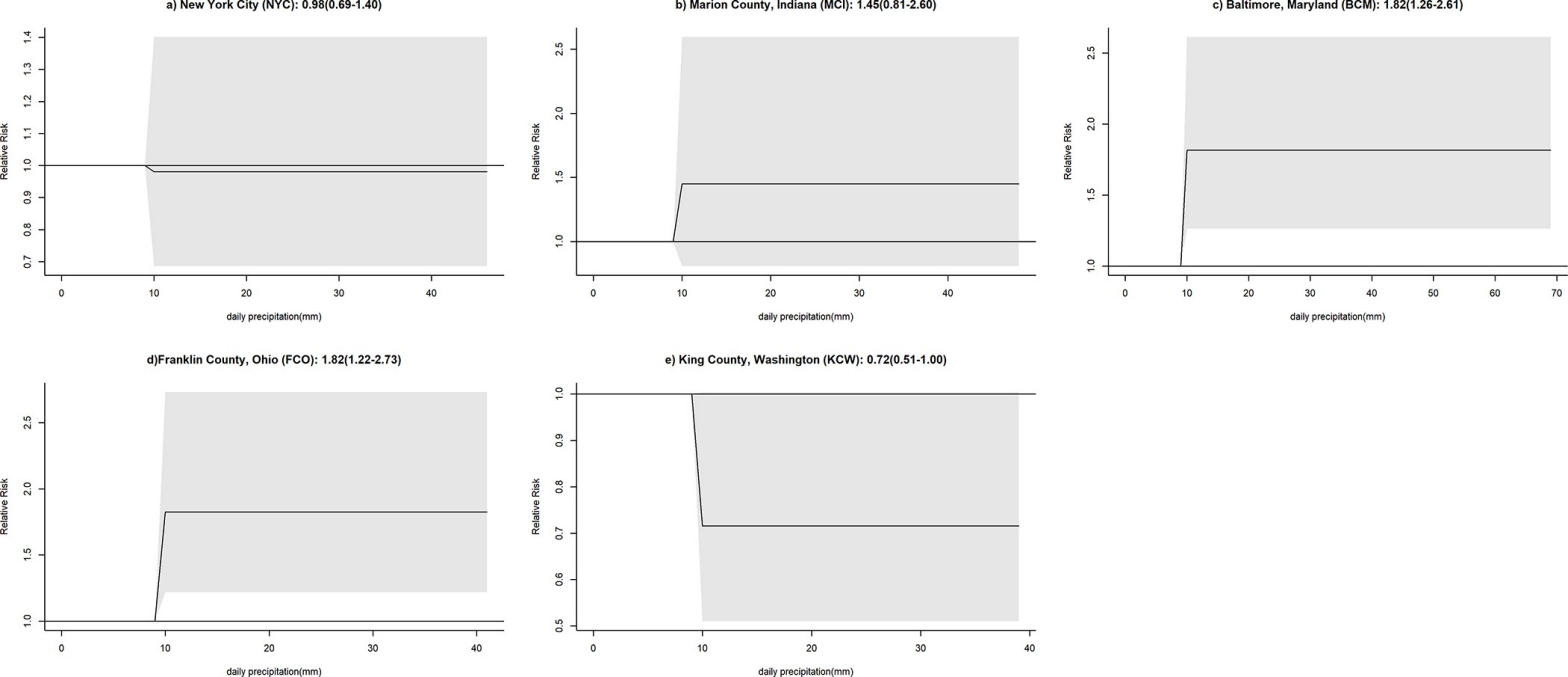
Effect of precipitation on COVID-19 infection risk with precipitation classified into two categories using a threshold of 10 mm. The model was adapted to estimate the risk of COVID-19 infection above 10 mm of precipitation relative to the risk of COVID-19 up to 10 mm of precipitation. Shaded areas represent 95% confidence intervals.

Analyses with all values included were then repeated using a precipitation threshold set at 10 mm with only two risk categories, one below and one above the threshold (Fig 9). The modified model resulted in altered QAIC values with most datasets showing slightly decreased values with only NYC showing an increase (NYC: 27587.95, MCI: 8134.17, BCM: 8212.48, FCO: 8106.83, KCW: 9507.42). For days of >10 mm of precipitation, the relative risk of COVID-19 infection was 0.98 (0.69–1.40) for NYC, 1.45 (0.81–2.60) for MCI, 1.82 (1.26–2.61) for BAL, 1.82 (1.22–2.73) for FCO, and 0.72 (0.51–1.00) for KCW. (Fig 9).

#### Relationship between cloud coverage and COVID-19 infection

**Fig 10:**
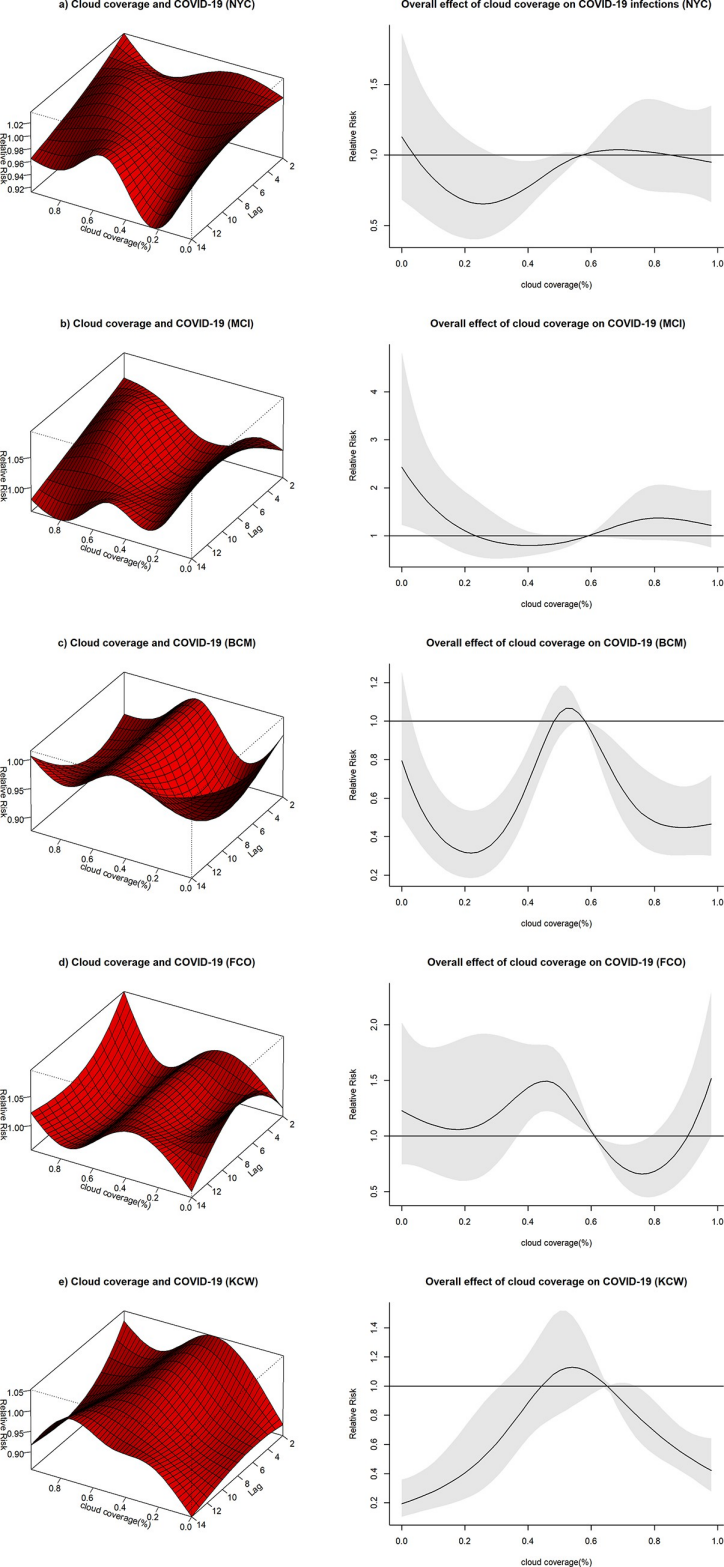
3D Plots with the effect of cloud coverage on COVID-19 along the lag period and overall effect plots of cloud coverage on COVID-19 infections. The surface of the 3D plots illustrates the relative risk of COVID-19 infection (y-axis) for different levels of cloud coverage (x-axis, range from 0–1) across different lag times (z-axis, days). The overall effect plots provide an estimate of the combined relative risk of COVID-19 across the 14 day lag period with cloud coverage represented on the x-axis and the relative risk on the y-axis.

The effect of cloud coverage on the risk of COVID-19 varied between study sites. For BCM and KCW, the relative risk of COVID-19 infection peaked shortly below the mean and then decreased again, indicating a lower risk of COVID-19 infection both for levels of low and high cloud coverage (Fig 10C and 10E). A similar pattern was observed for FCO (Fig 10D), though another peak in risk at high levels of cloud coverage was noted. Peaks in risks for BCM, FCO and KCW were found between cloud coverage levels of 46 to 54%. The effect of cloud coverage observed in NYC and MCI was less pronounced, especially when considering high levels of cloud coverage, though the two sites seemed to show a somewhat similar pattern (Fig 10A and 10B).

## Discussion

In the present study, higher than average temperature was shown to be consistently associated with a reduced risk of COVID-19 transmission for four out of the five study sites. Lower than average temperatures were associated with an increased risk of COVID-19 at least within an intermediate temperature range at four study sites. This range varied considerably and, with the exception of NYC, was followed by a reduced risk of infection at the lowest range of temperatures. Higher than average humidity levels were found to be associated with a higher risk of COVID-19 infection in four out of five study sites, the effect was statistically significant for three study sites. Lower than average humidity levels seemed to have no effect on COVID-19 transmissions, with the exception of one study site (KCW). The strength of the effects of temperature and humidity varied by study site and were strongly influenced by each other. In NYC, the effect of temperature was more pronounced and obscured the effect of humidity. In BCM, the effect of humidity was more pronounced, obscuring the effect of temperature. The effect of precipitation on COVID-19 transmission was less uniform and seemed to vary, depending on the study site. Medium range cloud coverage (46 to 54%) was associated with an increased risk of COVID-19 infection at three study sites.

The results of this study are based on more than a full year of COVID-19 infection data which strengthens the study with respect to the observed range of meteorological parameters examined and study power. The focus on the temperate climate zone constrains the generalizability of the study on the one hand, but allows us to investigate the influence of climatic factors on COVID-19 infection risk more deeply, on the other hand. Confounding by seasonal change, day of the week, COVID-19-related federally imposed restrictions have been accounted for in the present study. The use of a DLNM allowed investigations of the overall and lag specific nonlinear effects of meteorological variables in COVID-19 transmission, which differentiates this study from most previous work with some exceptions. Runkle et al. also used a DLNM, but conducted their analysis early in the COVID-19 pandemic [9]. In many instances, the associations between temperature and humidity observed in this study showed pronounced similarities across the study sites. These similarities foster hypotheses about common causes underlying these relationships and suggests that the findings of the study are generalizable for temperate climatic zones within and possibly beyond the USA. Notwithstanding these similarities, inconsistencies in the associations between study sites -if not explained by actual differences between these study sites (e.g., behavioural/environmental)- might reflect limitations of the study. It should be noted that, despite the use of a case-crossover design and incorporation of multiple confounders, the possibility of residual confounding, for example by air pollutants or seasonality, remains a possibility. Beyond the possibility of residual confounding, effect modification by meteorological variables such as precipitation and cloud coverage might be relevant and should be investigated in future studies.

Consistent with the findings from this study, two early studies also found higher temperatures to be negatively associated and humidity positively associated with COVID-19 infections [6, 10]. Oliveiros et al. correlated COVID-19 doubling time with meteorological variables in Chinese provinces between January 23 to March 1, 2020. Luo et al. have correlated an estimated proxy of the reproductive number with meteorological variables from January 23 to February 10, 2020. They point out “that the observed patterns of COVID-19 are not completely consistent with the hypothesis that high absolute humidity may limit the survival and transmission of this new virus”. Another US-based study conducted by Runkle et al. at between March and April 2020 using a similar approach found evidence of a positive association between COVID-19 infections and humidity [9]. A recent in vitro analysis on the effect of temperature and humidity on the stability of SARS-CoV-2 observed that virus decay became markedly faster under increased temperatures. Across all temperature levels, virus decay under different humidity levels showed a U-shaped dependency with extreme humidity levels slowing virus decay (40% and 100% relative humidity compared to 65%) [35]. As humidity and temperature are closely correlated and disentanglement of their respective effects on COVID-19 necessitates a sufficient amount of data, this study, in comparison to previous studies, has the advantage of including data over a longer time period. In addition, the use of specific humidity in this study may have facilitated improvements in adjusted analysis due to reduced autocorrelation with temperature compared to absolute or relative humidity and temperature. The common hypothesis of wet and warm climate acting as a protective factor of COVID-19 infection is not supported by the findings of this study for the temperate climate zone. The implication is that a strictly seasonal pattern with increased COVID-19 infections in cold and dry winters and decreased infections in warm and wet summers does not seem very likely.

In the present study precipitation seemed to have a variable effect depending on the study site which became more evident when the threshold model was adopted. However further research is necessary to refine threshold levels and investigate underlying cause-effect relationships. Extreme precipitation events in temperate regions have been identified as a risk factor of influenza and the role of extreme precipitation events on COVID-19 should be investigated [36]. In the present study, the strongest daily precipitation events reflected as outliers in the data were associated with an increased risk of COVID-19 at some of the study sites. However, scarcity of extreme precipitation events in the current data resulted in a high level of uncertainty.

Cloud coverage is inversely correlated with the amount of solar radiation reaching earth [37]. Runkle et al. have used a similar approach to estimate the effect of solar radiation on COVID-19 in the early phase of the COVID-19 pandemic in select cities of the US including Seattle [9]. An association was found which highly resembles the hill-shaped association found for Seattle in this study. Solar radiation and cloud coverage might be so intricately linked that, in the absence of more mechanistic explanations, effects on COVID-19 infection risk might not be attributable to exclusively one or the other. As with influenza, solar radiation might adversely affect the survival of SARS-CoV-2 virus [38]. Transmission clusters are, however, strongly linked to indoor environments, diminishing the direct influence of solar radiation on viral breakdown [34]. An indirect causal link between cloud coverage and COVID-19 infection risk beyond the effect of solar radiation should therefore be considered and the findings of this study might rather reflect human behavioral changes influenced by cloud coverage.

## Conclusions

The findings of this study break with the assumption of wet and warm climate acting as a protective factor. It follows, that periods of warm and humid weather, as frequently observed over summer in some temperate regions, should not be generally considered a time of reduced risk of COVID-19 infections. Humidity does not seem to be associated with a decreased but an increased risk of COVID-19 infection in temperate climate settings. Rather, the effect of humidity and temperature seem to counterbalance each other to some extent resulting in an overall effect that might be positively or negatively associated with the risk COVID-19 infection. This overall effect seems to differ depending on study site, possibly favoring variable seasonal COVID-19 infection cycles. Temporal patterns of COVID-19 infections are still heavily influenced by many factors including social distancing interventions, the spread of new variants, and vaccination programs. It is therefore too early for a verdict on seasonal patterns of COVID-19 infection. However, the results of this study do not suggest that a general seasonal pattern with increased COVID-19 infections in the winter and decreased infections in the summer is to be expected due to weather factors. COVID-19 related health policy should therefore not be planned exclusively around an expected easing of case numbers in the summer and increases in the fall or winter.

The effects of cloud coverage and precipitation on COVID-19 found in this study do not allow for specific recommendations. Rather, some meteorological variables are likely to indirectly influence the risk of COVID-19 infection by modifying human behavior, but their effect might vary depending on location, which needs to be further investigated.

## Supporting information

S1 Fig Correlations between the meteorological variables in New York City with corresponding Spearman’s correlation coefficients. Correlations between meteorological variables (temperature, specific humidity, precipitation and cloud coverage) were assessed using Spearman’s correlation coefficient. Strong correlations were observed between temperature and specific humidity as well as cloud coverage and precipitation (Spearman’s correlation coefficient 0.92 and 0.65, respectively).
(TIFF)

S2 Fig Residual autocorrelation plots for precipitation model without adjustments and including lag/logged lag/lag residuals (lag1) to control for autocorrelation using the NYC dataset. Autocorrelation for a lag period of 20 days (x-axis) was first assessed without further adjustment (a) using the partial autocorrelation function (PACF). Autocorrelation was strongest at lag of day 1. Including the COVID-19 case count of the previous day (Yt-1) (b) or the logged COVID-19 case count of the previous day (log(Yt-1)) (c) resulted in comparable reductions of autocorrelation among model residuals. Inclusion of lagged residuals of daily case count of day 1 (d) also led to some reduction of autocorrelation but the effect was considerably less pronounced.
(TIFF)

S3 Fig Residual autocorrelation plots for cloud coverage model without adjustments and including lag/logged lag/lag residuals (lag1) to control for autocorrelation using the NYC dataset. Autocorrelation for a lag period of 20 days (x-axis) was first assessed without further adjustment (a) using the partial autocorrelation function (PACF). Autocorrelation was strongest at lag of day 1. Including the COVID-19 case count of the previous day (Yt-1) (b) or the logged COVID-19 case count of the previous day (log(Yt-1)) (c) resulted in comparable reductions of autocorrelation among model residuals. Inclusion of lagged residuals of daily case count of day 1 (d) also led to some reduction of autocorrelation but the effect was considerably less pronounced.
(TIFF)

S4 Fig 3D Plots with the effect of precipitation on COVID-19 along the lag period and overall effect plots of precipitation on COVID-19 infections. The surface of the 3D plots illustrates the relative risk of COVID-19 infection (y-axis) for different levels of precipitation (x-axis, millimeters) across different lag times (z-axis, days). The overall effect plots provide an estimate of the combined relative risk of COVID-19 across the 14-day lag period with precipitation levels represented on the x-axis and the relative risk on the y-axis.
(TIFF)

S1 Table QAIC values returned for varying combinations of degrees of freedom for meteorological variables and lag periods using the NYC dataset. Model fit for different combinations of degrees of freedom for each meteorological parameter and lag periods were assessed to optimize model fit of the distributed lag nonlinear model. The lowest absolute QAIC value retrieved for a given combination was considered as the combination resulting in the best model fit.
(DOCX)
