# Supplementary figures and images for: The effect of temperature, humidity, precipitation and cloud coverage on the risk of COVID-19 infection in temperate regions of the USA—A case-crossover study
Source: PLoS One. 2022 Sep 15;17(9):e0273511. doi: 10.1371/journal.pone.0273511 (PMC9477315; doi:10.1371/journal.pone.0273511)

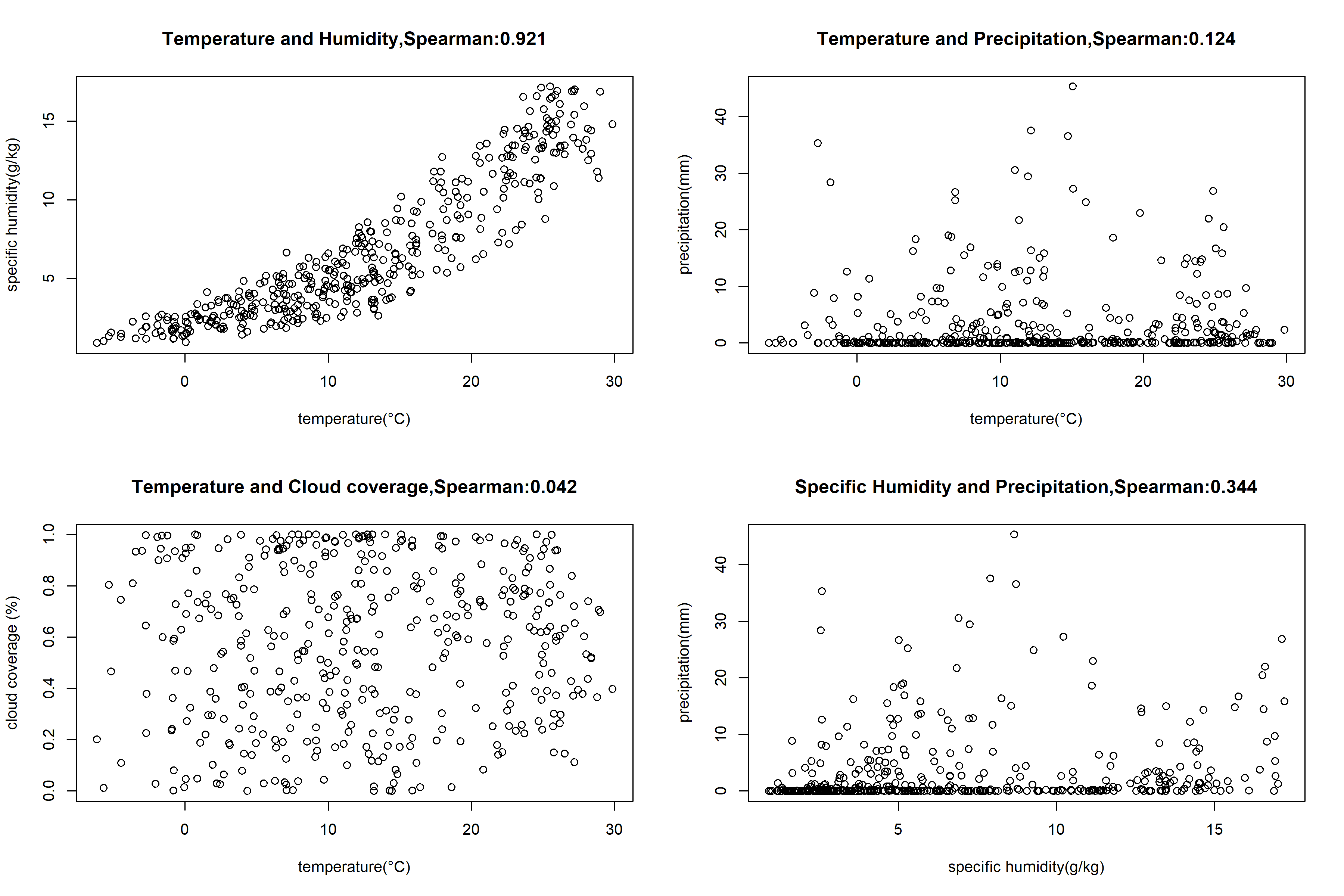

Supplement: S1 Fig — Correlations between meteorological variables (temperature, specific humidity, precipitation and cloud coverage) were assessed using Spearman’s correlation coefficient. Strong correlations were observed between temperature and specific humidity as well as cloud coverage and precipitation (Spearman’s correlation coefficient 0.92 and 0.65, respectively). (TIFF) [file pone.0273511.s001.tiff]

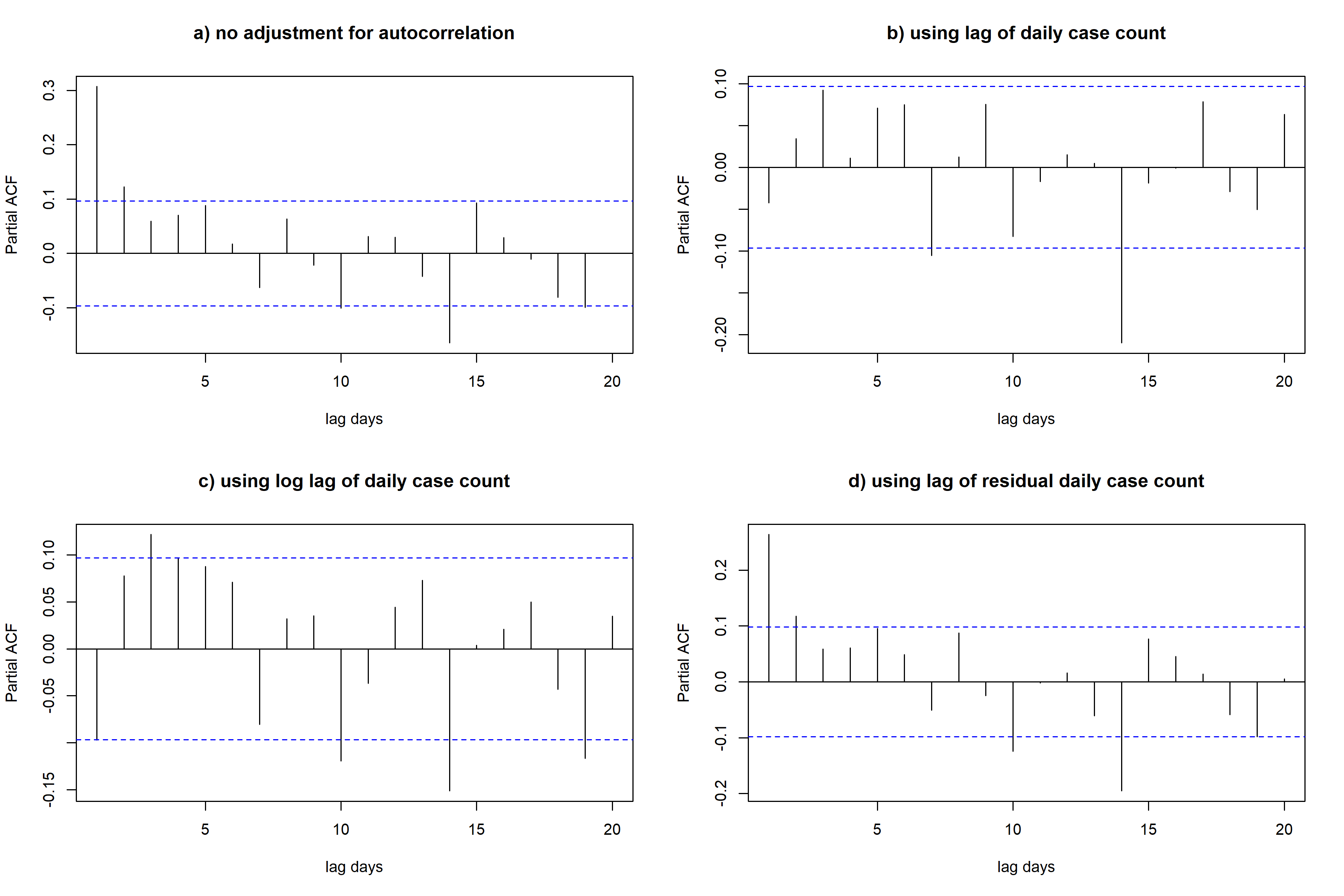

Supplement: S2 Fig — Autocorrelation for a lag period of 20 days (x-axis) was first assessed without further adjustment (a) using the partial autocorrelation function (PACF). Autocorrelation was strongest at lag of day 1. Including the COVID-19 case count of the previous day (Yt-1) (b) or the logged COVID-19 case count of the previous day (log(Yt-1)) (c) resulted in comparable reductions of autocorrelation among model residuals. Inclusion of lagged residuals of daily case count of day 1 (d) also led to some reduction of autocorrelation but the effect was considerably less pronounced. (TIFF) [file pone.0273511.s002.tiff]

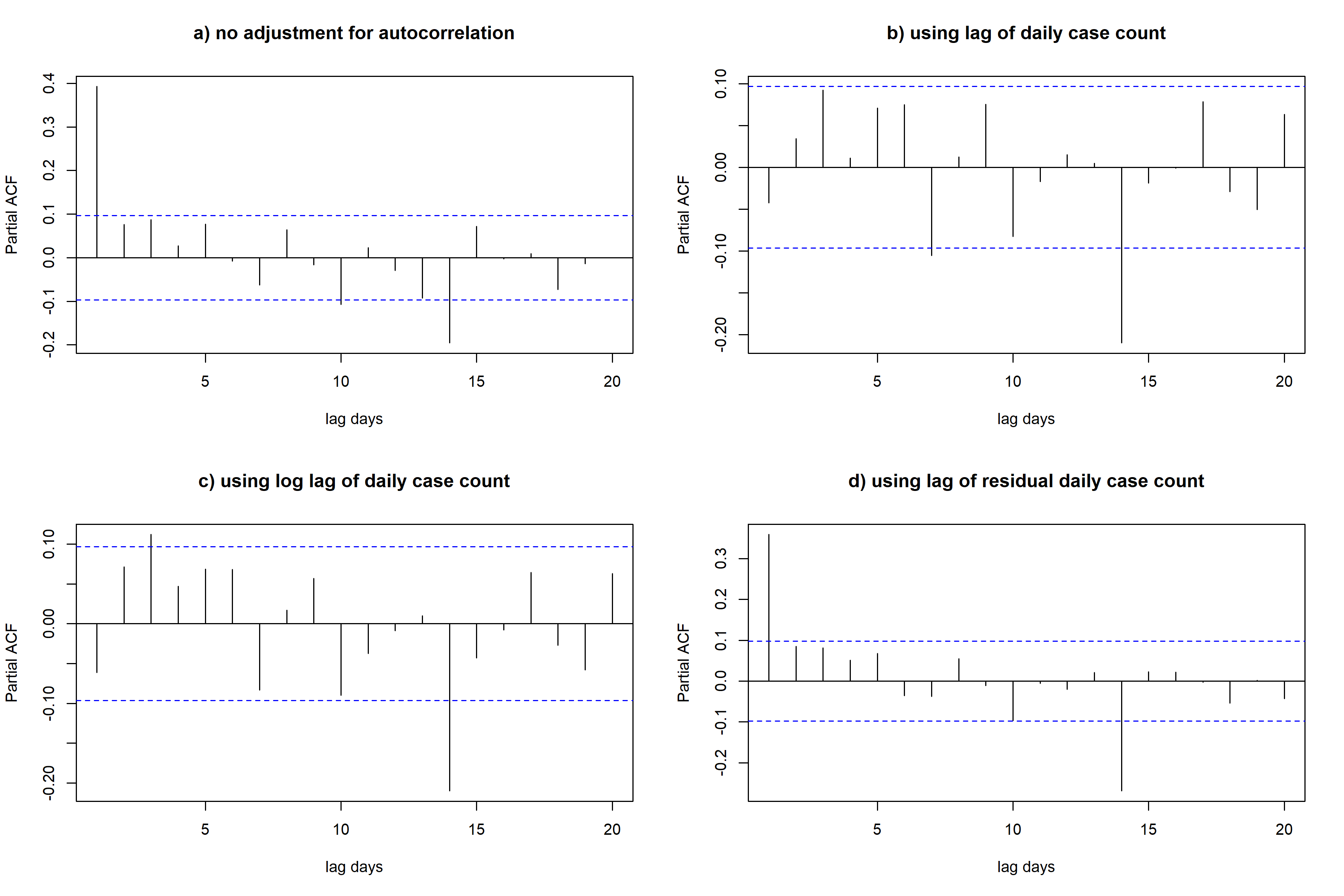

Supplement: S3 Fig — Autocorrelation for a lag period of 20 days (x-axis) was first assessed without further adjustment (a) using the partial autocorrelation function (PACF). Autocorrelation was strongest at lag of day 1. Including the COVID-19 case count of the previous day (Yt-1) (b) or the logged COVID-19 case count of the previous day (log(Yt-1)) (c) resulted in comparable reductions of autocorrelation among model residuals. Inclusion of lagged residuals of daily case count of day 1 (d) also led to some reduction of autocorrelation but the effect was considerably less pronounced. (TIFF) [file pone.0273511.s003.tiff]

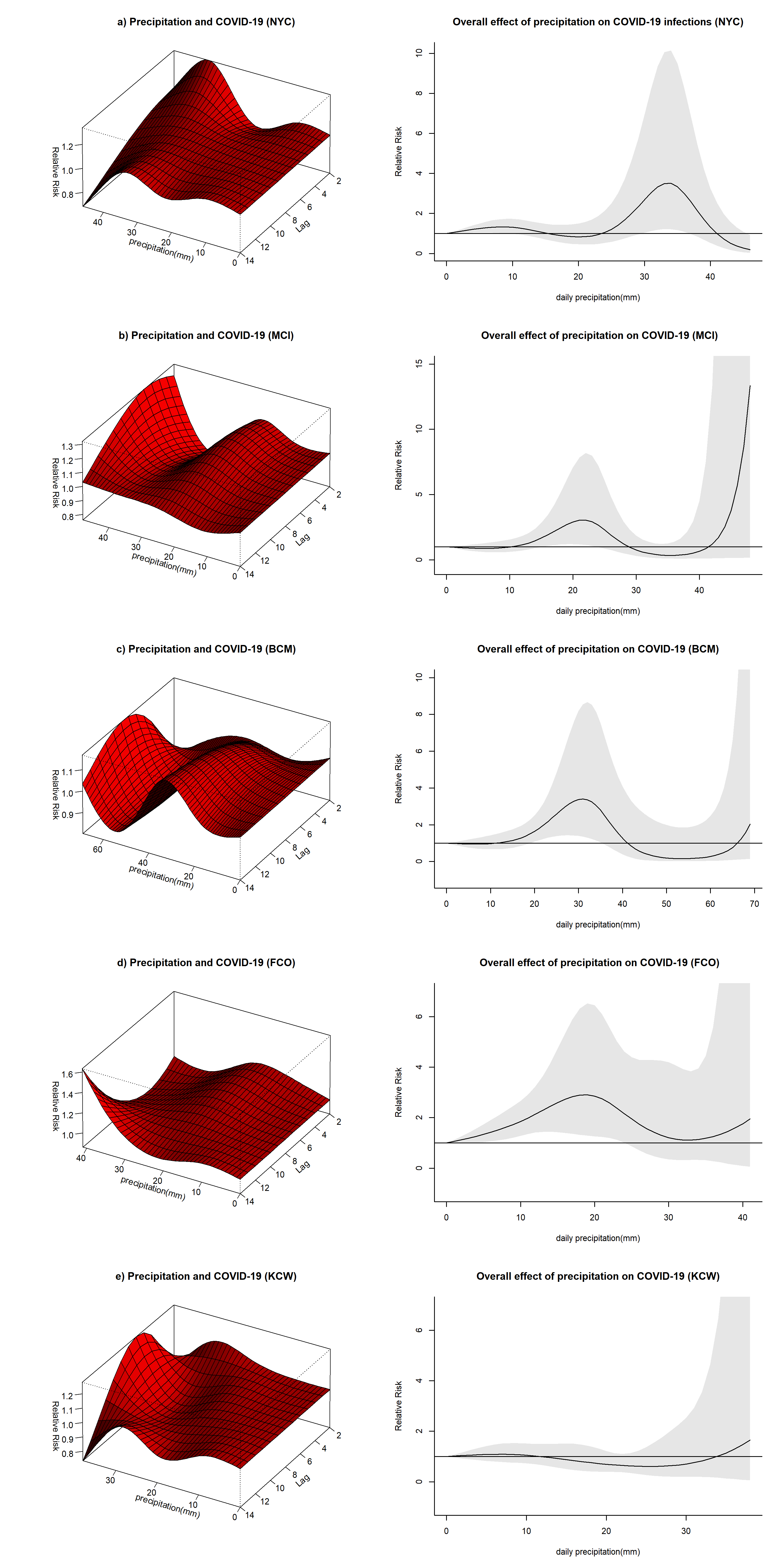

Supplement: S4 Fig — The surface of the 3D plots illustrates the relative risk of COVID-19 infection (y-axis) for different levels of precipitation (x-axis, millimeters) across different lag times (z-axis, days). The overall effect plots provide an estimate of the combined relative risk of COVID-19 across the 14-day lag period with precipitation levels represented on the x-axis and the relative risk on the y-axis. (TIFF) [file pone.0273511.s004.tiff]
