# Supplementary material for: The effect of temperature, humidity, precipitation and cloud coverage on the risk of COVID-19 infection in temperate regions of the USA—A case-crossover study
Source: PLoS One. 2022 Sep 15;17(9):e0273511. doi: 10.1371/journal.pone.0273511 (PMC9477315; doi:10.1371/journal.pone.0273511)
Supplement: S1 Table — Model fit for different combinations of degrees of freedom for each meteorological parameter and lag periods were assessed to optimize model fit of the distributed lag nonlinear model. The lowest absolute QAIC value retrieved for a given combination was considered as the combination resulting in the best model fit. (DOCX) [file pone.0273511.s005.docx]

|  | **df of meteorological variable** | | | | | |  |
| --- | --- | --- | --- | --- | --- | --- | --- |
|  | 3 | 4 | 5 | 6 | 7 | 8 |  |
| **lag(df:3)** |  |  |  |  |  |  |  |
| temperature | 27323.83 | 27156.22 | 27063.01 | 27304.38 | 27706.21 | 28068.67 |  |
| precipitation | 29246.03 | 27806.19 | 27797.16 | 26853.77 | 26689.49 | 26914.04 |  |
| humidity | 28154.73 | 28508.63 | 28865.04 | 28854.15 | 28607.35 | 28007.66 |  |
| cloud | 29842.05 | 29541.26 | 29316.49 | 29726.57 | 30087.06 | 30412.88 |  |
| **lag(df:4)** |  |  |  |  |  |  |  |
| temperature | 27604.44 | 27560.67 | 27441.42 | 27608.56 | 28081.31 | 28432.79 |  |
| precipitation | 29654.51 | 28195.64 | 28260.01 | 27407.01 | 27079.91 | 27187.57 |  |
| humidity | 28531.8 | 29058.17 | 29551.5 | 29634.02 | 29507.66 | 28962.25 |  |
| cloud | 29885.5 | 29381.12 | 28645.17 | 29089.01 | 29453.26 | 30041.92 |  |
| **lag(df:5)** |  |  |  |  |  |  |  |
| temp | 27403.95 | 27494.03 | 27460.93 | 27661.08 | 28377.64 | 28836.7 |  |
| precipitation | 29978.45 | 28694.69 | 28891.96 | 28130.33 | 27944.37 | 28190.5 |  |
| humid | 28555.45 | 29057.94 | 29761.55 | 29979.72 | 29948.63 | 29457.09 |  |
| cloud | 30046.18 | 29575.44 | 29008.35 | 29629.74 | 30085.59 | 30707.94 |  |
